# Microarray Expression Profiling of Long Non-Coding RNAs Involved in Nasopharyngeal Carcinoma Metastasis

**DOI:** 10.3390/ijms17111956

**Published:** 2016-11-23

**Authors:** Xin Wen, Xinran Tang, Yingqin Li, Xianyue Ren, Qingmei He, Xiaojing Yang, Jian Zhang, Yaqin Wang, Jun Ma, Na Liu

**Affiliations:** Sun Yat-sen University Cancer Center, State Key Laboratory of Oncology in South China, Collaborative Innovation Center of Cancer Medicine, Guangzhou 510060, China; wenxin1@sysucc.org.cn (X.W.); tangxr@sysucc.org.cn (X.T.); liyingq@sysucc.org.cn (Y.L.); renxy@sysucc.org.cn (X.R.); heqm@sysucc.org.cn (Q.H.); yangxiaoj@sysucc.org.cn (X.Y.); zhangjian@sysucc.org.cn (J.Z.); wangyaq@sysucc.org.cn (Y.W.)

**Keywords:** nasopharyngeal carcinoma, metastasis, long non-coding RNA, microarray

## Abstract

Increasing evidence has demonstrated a significant role for long non-coding RNAs (lncRNAs) in tumorigenesis. However, their functions in nasopharyngeal carcinoma (NPC) metastasis remain largely unknown. In this study, a model comparing high and low metastatic NPC cell lines (5-8F vs. 6-10B and S18 vs. S26) was constructed to determine the expression profile of lncRNAs using the microarray analysis, and we found 167 lncRNAs and 209 mRNAs were differentially expressed. Bioinformatic analysis indicated that the dysregulated mRNAs participated in important biological regulatory functions in NPC. Validation of 26 significantly dysregulated lncRNAs by qRT-PCR showed the expression patterns of 22 lncRNAs were in accordance with the microarray data. Furthermore, the expression level of ENST00000470135, which was the most upregulated lncRNA in high metastatic cell lines, was significantly higher in NPC cell lines and tissues with lymph node metastasis (LNM) and knocking down ENST00000470135 suppressed the migration, invasion and proliferation of NPC cells in vitro. In conclusion, our study revealed expression patterns of lncRNAs in NPC metastasis. The dysregulated lncRNAs may act as novel biomarkers and therapeutic targets for NPC.

## 1. Introduction

Nasopharyngeal carcinoma (NPC) is one of the leading head and neck malignancies and prevalent in Southeast China, Southeast Asia, the Middle East, Northeast Africa and Alaska [1,2,3,4,5]. Although local and regional control of NPC has been improved after the introduction of intensity-modulated radiation therapy and concurrent chemoradiotherapy, the prognosis of patients with distant metastasis still remains very poor [6,7]. Therefore, identification of the molecular mechanisms involved in the metastasis of NPC is urgently required to develop individualized treatment of this disease.

Metastasis is an important characteristic of malignant tumors and the leading cause of cancer-related deaths [8]. Recently, long non-coding RNAs (lncRNAs), which are defined as RNAs longer than 200 nucleotides without protein-coding potential [9], have been found to regulate cell growth, metastasis and apoptosis [10,11,12] in most tumor types via controlling transcriptional, post-transcriptional and/or epigenetic mechanisms [13,14,15]. To date, several studies have shown that a number of lncRNAs are involved in the progression of NPC, including NEAT1, HNF1A-AS, MALAT1, HOTAIR and LINC00312 [16,17,18,19,20]. However, despite this progress, the precise functions and mechanisms of lncRNAs in the metastasis of NPC still remain unclear.

Two sets of cell lines, 5-8F (high tumorigenic and metastatic potential) and 6-10B (low tumorigenic and metastatic potential), which were derived from the NPC cell line SUNE-1 [21], and S18 (high tumorigenic and metastatic potential) and S26 (low tumorigenic and metastatic potential), which were derived from CNE-2 cells [22,23], represent good models for investigating the metastasis of NPC. For the first time, we compared these high metastatic potential and low metastatic potential cell lines (5-8F vs. 6-10B and S18 vs. S26) using microarrays to identify dysregulated lncRNAs that participate in NPC tumorigenesis. Gene Ontology (GO) and pathway analysis was performed to better understand the differentially expressed mRNAs. Subsequently, we validated the results via quantitative reverse transcription polymerase chain reaction (qRT-PCR) and selected the most differentially expressed lncRNA ENST00000470135, which had previously not been reported in order to clarify its biological function. Our research provides a new insight into metastasis of nasopharyngeal carcinoma.

## 2. Results

### 2.1. Profiles of Differentially Expressed Long Non-Coding RNAs (lncRNAs) and mRNAs

**Figure 1:**
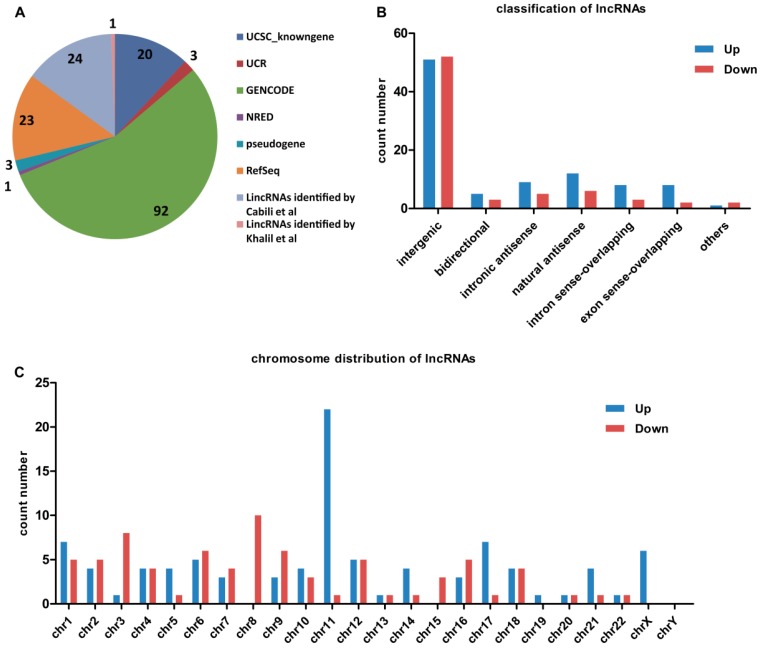
Distribution of aberrantly expressed long non-coding RNAs (lncRNAs) in the high metastatic potential nasopharyngeal carcinoma (NPC) cell lines when compared with low metastatic potential NPC cell lines. (**A**) pie chart showing the relative numbers of lncRNAs based on the most authoritative databases; (**B**) distribution of dysregulated lncRNAs according to their classification; and (**C**) lncRNA profiles on human chromosomes. The number of deregulated species, including detailed upregulated and downregulated lncRNAs are shown. chr: chromosome.

We conducted lncRNA and mRNA expression profiling of two groups of cell lines (5-8F vs. 6-10B and S18 vs. S26) via microarray analysis. In total, 167 lncRNAs (94 upregulated and 73 downregulated; Table S1) and 209 mRNAs (162 upregulated and 47 downregulated; Table S2) were found to be differentially expressed in high metastatic cells when compared with low metastatic cells (fold change ≥2 in both groups, normalized intensity of at least one cell line of each group ≥5). The lncRNAs are collected from highly respected databases, including Gencode, RefSeq, UCSC Knowngene and five other high quality publications (Figure 1A). To clarify the expression signatures of the dysregulated lncRNA, we analyzed upregulated or downregulated lncRNAs identified in high metastatic potential cell lines according to their classification and chromosome distribution (Figure 1B,C).

### 2.2. GO and KEGG Pathway Analysis

**Figure 2:**
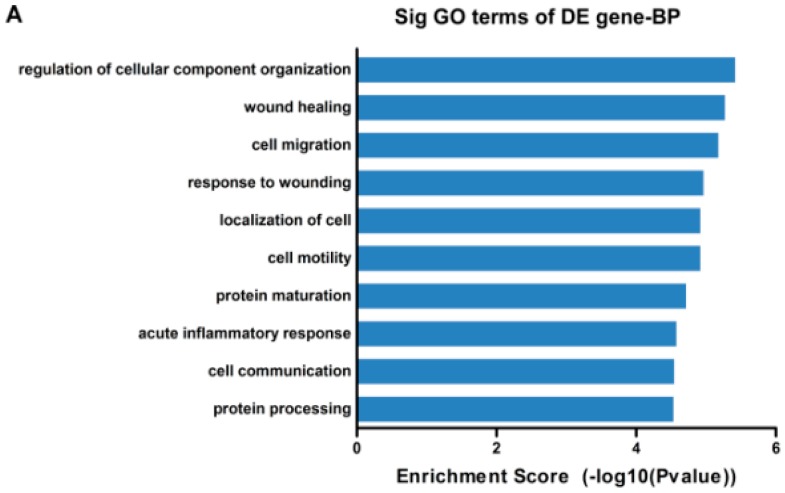

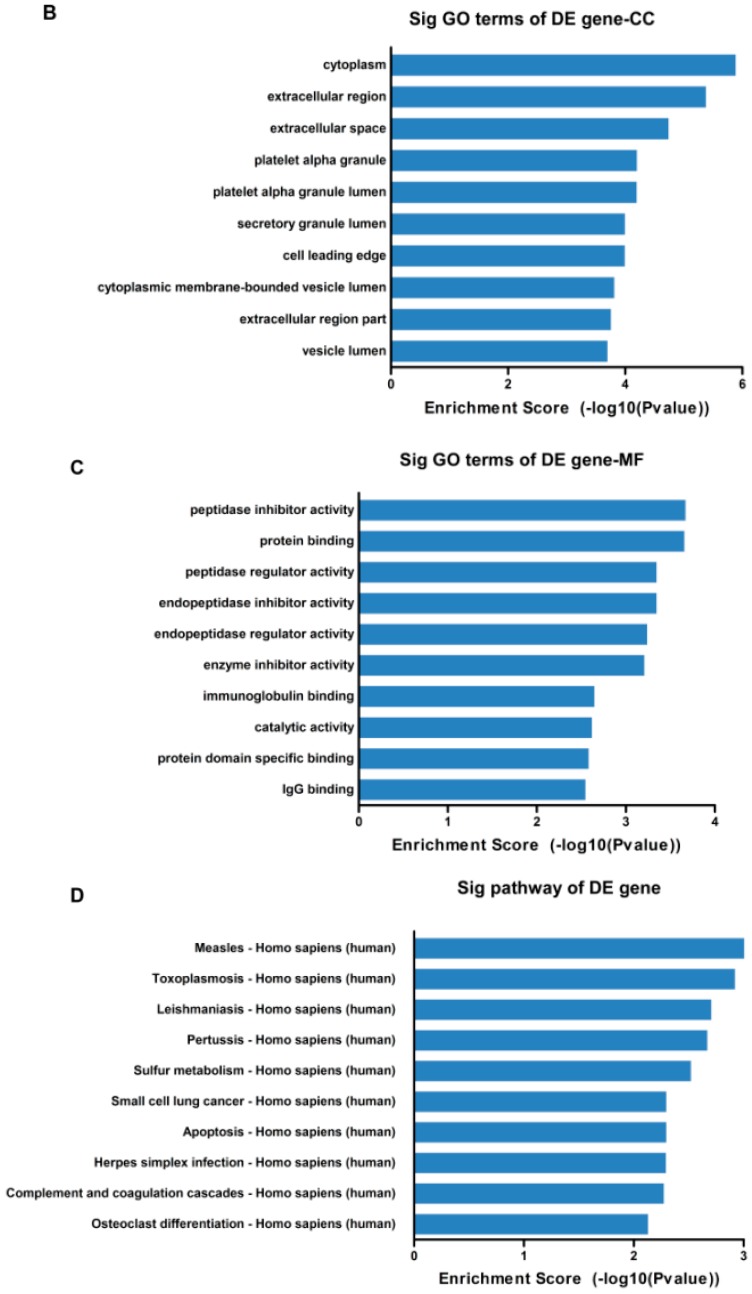
Gene ontology (GO) and pathway analysis of dysregulated genes in high metastatic potential NPC cell lines when compared with low metastatic potential NPC cell lines. (**A**–**C**) the top ten enrichment score (−log10 (*p*-value)) values for significantly enriched GO terms including Biological Process (**A**), Cellular Component (**B**) and Molecular Function (**C**), and (**D**) the top ten highest and significant pathways. *p*-Values were calculated using the significance test (**A**–**C**) and Fisher’s exact test (**D**).

To explore the potential function of the differentially expressed mRNAs in high metastatic cells when compared with low metastatic cells, GO analysis was performed to describe biological process (BP), cellular component (CC) and molecular function (MF; Tables S3–S5). The GO terms were determined by calculating Enrichment Score (*p* < 0.05) as previously described [24]. In our study, the aberrantly expressed genes were mainly enriched for GO terms related to regulation of cellular component organization, wound healing and cell migration involved in biological process, and cytoplasm, extracellular region and extracellular space linked with cellular component, as well as peptidase inhibitor activity, protein binding and peptidase regulator activity in molecular function. The top ten highest and most significant GO terms are shown in Figure 2A–C.

Pathway analysis based on the Kyoto Encyclopedia of Genes and Genomes (KEGG) database was used, and we identified a total of 26 pathways with significant differences (*p* < 0.05) in gene expression between the high metastatic potential and low metastatic potential cell lines (Table S6). The pathway terms of top ten highest Enrichment Scores are shown in Figure 2D; a number of these pathways, including the apoptosis pathway and small cell lung cancer pathway, are associated with carcinogenesis.

### 2.3. Validation of Significantly Dysregulated lncRNAs by qRT-PCR

**Figure 3:**
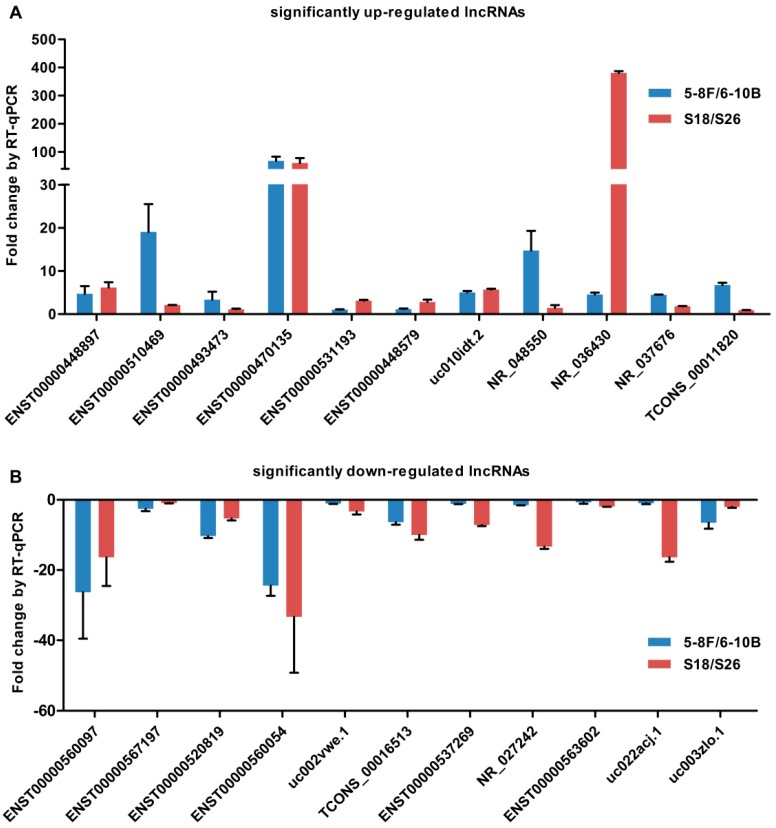
Validation of significantly dysregulated lncRNAs by qRT-PCR. The figure shows the expression patterns of 22 lncRNAs including 11 upregulated (**A**) and 11 downregulated (**B**) were consistent with the microarray data.

Among the aberrantly expressed lncRNAs, 26 were significantly dysregulated (fold change >5 in both groups; Table 1). In order to verify the microarray data, we selected the 26 most significantly dysregulated lncRNAs (fold change >5 in both groups), which included 15 upregulated lncRNAs and 11 downregulated lncRNAs and then validated their expression level by quantitative RT-PCR (qRT-PCR) in two sets of NPC cells (5-8F vs. 6-10B and S18 vs. S26). The results showed that the expression patterns of 22 lncRNAs were consistent with the microarray data (Figure 3A,B), which demonstrated the reliability of the microarray data. Among the 22 validated lncRNAs, the most differentially expressed lncRNA was ENST00000470135 (fold change >60 in both groups).

### 2.4. ENST00000470135 Is Upregulated in Nasopharyngeal Carcinoma (NPC) Cells and Tissues with Lymph Node Metastasis

**Figure 4:**
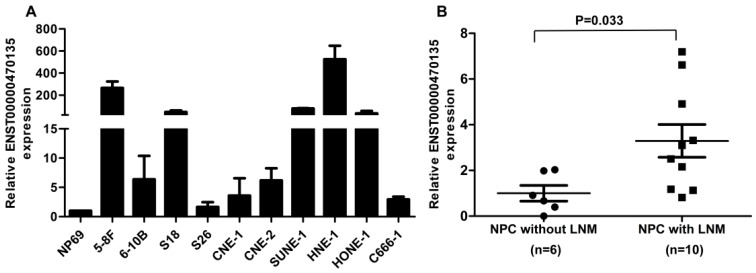
ENST00000470135 is upregulated in NPC cell lines and tissues with lymph node metastasis (LNM). (**A**) relative expression of ENST00000470135 in immortalized nasopharyngeal epithelial cell line NP69 and NPC cell lines; and (**B**) relative expression of ENST00000470135 in NPC tissues from patients without LNM (*n* = 6) and patients with LNM (*n* = 10). Values are mean ± SD; *p*-values were calculated using the Student’s *t*-test.

To validate the importance of ENST00000470135 in NPC, we firstly examined the expression levels of ENST00000470135 in the immortalized nasopharyngeal epithelial cell line NP69 and ten NPC cell lines using qRT-PCR. The RNA level of ENST00000470135 was remarkably higher in all of the NPC cell lines tested (Figure 4A). Moreover, we analyzed the expression of ENST00000470135 in 16 freshly frozen NPC tissues (six without lymph node metastasis (LNM) and 10 with LNM), and found that ENST00000470135 was significantly upregulated in tumors from patients with lymph node metastasis compared to those from patients without lymph node metastasis (Figure 4B; *p* = 0.033). These results strongly suggest that ENST00000470135 is upregulated in NPC.

### 2.5. Depletion of ENST00000470135 Has Significant Effect on NPC Cell Migration, Invasion and Proliferation In Vitro

**Figure 5:**
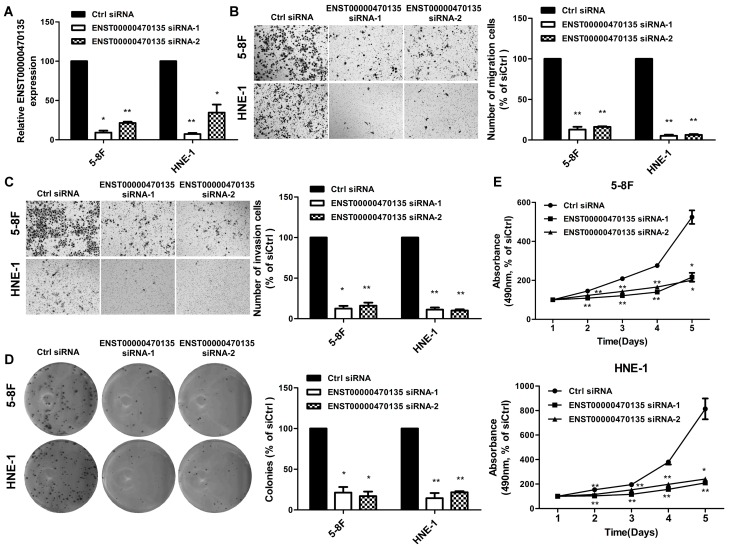
Effects of ENST00000470135 depletion on NPC cell migration, invasion and proliferation in vitro. (**A**) siRNA targeting ENST00000470135 significantly knocked down the expression of ENST00000470135 in 5-8F and HNE-1 cells; (**B**,**C**) representative images (**left**) and quantification (**right**) of the Transwell migration assay (**B**) and Transwell invasion assay (**C**) of 5-8F and HNE-1 cells transfected with ENST00000470135 siRNA or Ctrl siRNA; (**D**) representative images (**left**) and quantification (**right**) of the colony formation assay of 5-8F and HNE-1 cells transfected with ENST00000470135 siRNA or Ctrl siRNA; and (**E**) representative images of the 3-(4,5)-dimethylthiahiazo(-z-y1)-3,5-di-phenytetrazoliumromide (MTT) assay of 5-8F (**upper**) and HNE-1 (**lower**) cells transfected with ENST00000470135 siRNA or Ctrl siRNA. Values are mean ± SD; * *p* < 0.05, ** *p* < 0.001 compared with control using the Student’s *t*-test.

To assess whether aberrant expression of ENST00000470135 affects the motility and invasion ability of NPC cells, 5-8F and HNE-1 cells were transiently transfected with siRNA targeting ENST00000470135 or Ctrl siRNA (Figure 5A). In the Transwell migration and invasion assays, the migratory and invasive ability of 5-8F and HNE-1 cells transfected with ENST00000470135 siRNA was significantly lower than negative control cells (Figure 5B,C; * *p* < 0.05, ** *p* < 0.001). The results suggest that the knockdown of ENST00000470135 dramatically suppresses the migration and invasion of NPC cells.

Colony formation assay and 3-(4,5)-dimethylthiahiazo (-z-y1)-3,5-di-phenytetrazoliumromide (MTT) assay were performed to further explore whether depletion of ENST00000470135 affects the viability and proliferation of NPC cells. The colony formation rate was significantly lower in 5-8F and HNE-1 cells transiently transfected with ENST00000470135 siRNA than in cells transfected with respective control (Figure 5D; * *p* < 0.05, ** *p* < 0.001). Moreover, 5-8F and HNE-1 cells transfected with ENST00000470135 siRNA displayed significant growth inhibition (Figure 5E; * *p* < 0.05, ** *p* < 0.001). These in vitro biological assays indicate that depletion of ENST00000470135 decreases the ability of NPC cells to migrate, invade and proliferate, providing strong evidence that ENST00000470135 may promote carcinogenesis and metastasis in NPC and further confirming the reliability of the microarray data.

## 3. Discussion

The highest incidence of nasopharyngeal carcinoma worldwide is observed in Southern Asia; the age-standardized incidence per 100,000 males varies from 20–50 in southern China to 0.5 in white populations [1,25,26]. The remarkable geographic and racial distribution of NPC indicates that the pathogenesis and development of this cancer may be associated with genetic factors. It is well recognized that distant metastasis is the major pattern of failure in NPC. In this study, we established a model to compare high metastatic potential cell lines with low metastatic potential cell lines (5-8F vs. 6-10B and S18 vs. S26) in order to further explore the mechanisms that regulate metastasis in NPC.

Increasing evidence shows that lncRNAs play important roles in carcinogenesis, tumor progression and metastasis [11,27]. In our study, high-throughput microarray analysis was used to profile aberrantly expressed lncRNAs and mRNAs that might be involved in NPC metastasis and 167 lncRNAs and 209 mRNAs were found to be dysregulated. Recently, several studies confirm that some lncRNAs identified in other cancer types are also involved in NPC. Li et al. [28] showed that H19 (a long non-coding RNA) expression was significantly upregulated in NPC tissues and cell lines. Additionally, they found that H19 promoted invasion of NPC cells via the miR-630/EZH2 pathway. Jin et al. [18] concluded that upregulation of MALAT1 in NPC increased radioresistance by modulating miR-1/slug axis. Bo et al. [29] showed that AFAP1-AS1 promoted NPC cell metastasis via regulation of actin filament integrity. There are also several studies that have clarified the expression patterns of lncRNAs in NPC focusing on paired analyses of different types of tissues [30,31,32]. However, few studies have explored the relationships between dysregulated expression of lncRNAs and metastasis in NPC, especially using models based on cell lines.

GO analysis predicted that the dysregulated mRNAs were linked to biological process, cellular component and molecular function in NPC. The GO terms such as cell migration and cell motility involved in biological process indicated the associated gene product contributes to the metastasis of NPC. The results of pathway analysis showed that aberrantly expressed mRNAs were related to 26 signalling pathways in NPC. The correlation between these pathways and multiple diseases including NPC has been proved by previous studies. Among which, apoptosis [33,34,35], p53 [36,37] and NF-κB [38,39] have been reported to be closely related to NPC pathogenesis.

In validation of the reliability of the microarray data, strong correlations were observed between the expression levels of representative lncRNAs in both the microarray and qRT-PCR analyses. To confirm whether the lncRNAs identified by the microarray contribute to the progression of NPC, we explored the biological function of ENST00000470135, the most aberrantly expressed lncRNA. ENST00000470135 is a non-coding RNA from Ensembl [40] with a length of 673 bp, and it is spliced from RP5-884M6.1. This lncRNA has not been reported in the literature, so its biological function and regulation mechanism in nasopharyngeal carcinoma is still unclear. In this study, we found that ENST00000470135 was expressed at a significantly higher level in the NPC cell lines than the nasopharyngeal epithelial cell line NP69. Additionally, the expression of this lncRNA was upregulated in NPC tissues taken from patients with LNM compared to those from patients without LNM. Furthermore, in vitro functional experiments, including the Transwell, colony formation and MTT assays, indicated that knocking down ENST00000470135 suppresses the migration, invasion and proliferation of NPC cells. Above all, these results suggest that ENST00000470135 plays an important role in NPC metastasis, which is consistent with the findings of the microarray analysis.

Additionally, among the various kinds of biomarkers, circulating lncRNA is ideal due to its accessibility and noninvasiveness. Recent studies have demonstrated that circulating lncRNAs could forecast prognosis and predict therapeutic efficacy in different kinds of cancers [41,42,43]. For example, three circulating lncRNAs (LincRNA-p21, GAS 5, HOTAIR) have been discovered as biomarkers to predict chemoradiotherapy sensitivity in head and neck cancers including nasopharyngeal carcinoma [44], indicating the important value of circulating lncRNAs in clinical outcome predictions.

## 4. Materials and Methods

### 4.1. Cell Culture and Clinical Specimens

Eight human NPC cell lines 5-8F, 6-10B, CNE-1, CNE-2, SUNE-1, HNE-1, HONE-1 and C666-1 were maintained in RPMI-1640 (Gibco, Life Technologies, Carlsbad, CA, USA) supplemented with 10% fetal bovine serum (FBS; Gibco). Two NPC cell lines, S18 and S26, were maintained in Dulbecco’s modified Eagle’s medium (DMEM, Gibco) supplemented with 10% FBS (Gibco). The human immortalized nasopharyngeal epithelial cell line NP69 was cultured in keratinocyte/serum-free medium (Gibco) supplemented with bovine pituitary extract (BPE; Gibco) and epidermal growth factor (EGF, human recombinant; Gibco). The human immortalized nasopharyngeal epithelial cell line, NP69, and 8 human NPC cell lines (5-8F, 6-10B, CNE-1, CNE-2, SUNE-1, HNE-1, HONE-1 and C666-1), which had been authenticated, were generously provided by Musheng Zeng (Sun Yat-sen University Cancer Center, Guangzhou, China). Two NPC cell lines, S18 and S26, were obtained from Chaonan Qian (Sun Yat-sen University Cancer Center).

A total of 16 freshly frozen NPC tissue samples (from 10 patients with LNM and 6 patients without LNM) were collected at Sun Yat-sen University Cancer Center. Written informed consent was obtained from each patient before biopsy. This study was approved by the Institutional Ethical Review Board of our Cancer Center (GZR2016-108, 23 February 2016).

### 4.2. RNA Extraction and Quality Control

Total RNA was extracted from the 16 frozen NPC tissue specimens and 11 cell lines using TRIzol reagent (Invitrogen, Grand Island, NY, USA) according to the manufacturer’s instructions. The quality and amount of RNA were assessed using a NanoDrop ND-2000 spectrophotometer (Thermo Scientific, Rockford, IL, USA) and RNA integrity was assessed by standard denaturing agarose gel electrophoresis. Isolated RNAs were stored at −80 °C prior to lncRNAs microarray analysis and quantitative reverse transcription PCR.

### 4.3. Microarray Analysis

Arraystar Human LncRNA Microarray V3.0 (ArrayStar, Rockville, MD, USA) which contains 30,586 lncRNAs and 26,109 coding transcripts was performed to detect the genome-wide profiling compared the high metastatic potential (5-8F and S18) and low metastatic potential cell lines (6-10B and S26). The lncRNAs were collected from the majority of landmark public databases (Gencode, Refseq, ect.) as well as high-quality publications. In order to recognize individual transcripts accurately, we used a splice junction or specific exon probe to represent each transcript.

mRNA was purified with the extraction of rRNA according to the manufacturer’s instructions (mRNA-ONLYTM eukaryotic mRNA Isolation Kit, Epicentre, Madison, WI, USA). Each of the samples was amplified and transcribed into fluorescent cRNA without 3′ bias. After the labeled cRNAs were measured by NanoDrop ND-1000, blocking agent and fragmentation buffer were added. Then, the mixture was heated and the cRNA was diluted using the hybridization buffer. Finally, the labeled cRNAs were assembled to the human lncRNA array version 3.0 (8 × 60 K; Arraystar, Rockville, MD, USA). After washing and fixation, the hybridized arrays were scanned using an Agilent DNA scanner G2505C (Agilent Technologies, Santa Clara, CA, USA). KangChen Bio-tech (Shanghai, China) perfomed the microarray experiments.

### 4.4. Bioinformatics Data Analysis and Data Mining

Acquired array images were analyzed by Agilent Feature Extraction software (v11.0.1.1). GeneSpring GX v12.1 software package (Agilent Technologies, Santa Clara, CA, USA) was used to perform quantile normalization and subsequent data processing. Differentially expressed lncRNAs and mRNAs between the high and low metastatic potential cell lines (5-8F vs. 6-10B and S18 vs. S26) were identified through fold change and normalized intensities filtering (fold change ≥2 in both groups, normalized intensity of at least one cell line of each group ≥5). We have deposited the microarray data in the National Center for Biotechnology Information’s Gene Expression Omnibus (GSE89804).

The aberrantly expressed mRNAs in NPC metastasis were selected to perform GO and KEGG pathway anaysis. For GO analysis (Available online: http://geneontology.org/), the corresponding genes were divided into 3 classifications by enrichment analysis, including biological process (BP), cellular component (CC) and molecular function (MF). Using the latest KEGG (Available online: http://www.genome.jp/kegg/), we analyzed the differentially expressed mRNAs and calculated the enrichment of different pathways.

### 4.5. Reverse Transcription and Quantitative Real-Time RT-PCR

To measure the expression of selected lncRNAs, we firstly performed reverse transcription using random primers (Promega, Madison, WI, USA) and Moloney Murine Leukemia Virus (M-MLV) reverse transcriptase (Promega, Madison, WI, USA) and stored the RT products briefly at 4 °C or at −20 °C until use. Then, quantitative RT-PCR was conducted on a CFX96 Touch sequence detection system (Bio-Rad, Hercules, CA, USA) using SYBR Green (Platinum SYBR Green qPCR SuperMix-UDG reagents; Invitrogen, Grand Island, NY, USA). *GAPDH* was used as the normalization control and the relative expression levels were calculated using the 2^−ΔΔ*C*t^ method [45]. The real-time RT-PCR primers for the lncRNAs and *GAPDH* are shown in Table S7.

### 4.6. Oligonucleotide Transfection

The 5-8F or HNE-1 cells were seeded into 6-well plates 24 h before transfection. ENST00000470135-silencing oligonucleotides (ENST00000470135 siRNA) (sequences: siRNA-1 5′-GCAGCUCACAUGCCAGAAATT-3′; siRNA-2 5′-GUCUGGAAGUCUGCUCACATT-3′) or the negative control (Ctrl siRNA; GenePharma, Shanghai, China) were transfected into the cells using Lipofectamine™ 2000 (Invitrogen) at a final concentration of 100 nmol/L. The cells were harvested for the specified assays 24–48 h after transfection.

### 4.7. Transwell Migration and Invasion Assays

Cell migration and invasion ability were assessed using Transwell chambers (8-μm pores; Corning) coated with or without Matrigel (BD Biosciences, San Diego, CA, USA). The plates were placed in the culture incubator for at least 30 min at 37 °C, and then 5-8F or HNE-1 cells (5 × 10^4^ or 1 × 10^5^) were transfected and suspended in 200 μL serum-free medium and placed into the upper chambers. The lower chambers were filled with 500 μL medium supplemented with 10% FBS. After incubation for 12–24 h, the NPC cells that had migrated or invaded through the Matrigel and pores were fixed with 4% paraformaldehyde, stained using 0.5% crystal violet and manually counted using an inverted microscope (100×).

### 4.8. MTT Assay and Colony Formation Assay

For the 3-(4,5)-dimethylthiahiazo(-z-y1)-3,5-di-phenytetrazoliumromide (MTT) assay, 5-8F and HNE-1 cells transfected with siRNAs were seeded at a density of 1000 cells per well in 96-well plates. At 1, 2, 3, 4 and 5 days, an ELX800 spectrophotometric plate reader (Bio-Tek, Winooski, VT, USA) was used to measure the cell viability at 490 nm. For the colony formation assay, the transfected cells were plated at 400 cells per well in 6-well plates and cultured in the cell culture incubator for 7 to 12 days at 37 °C, fixed with 4% paraformaldehyde, stained using 0.5% crystal violet, and then the numbers of colonies were counted.

### 4.9. Statistical Analysis

Significance test was used for GO analysis and Fisher’s exact test was used for pathway analysis. For qRT–PCR validation analysis and functional analysis, the Student’s *t*-test was used to determine the significance of the differences between two groups. *p*-Values < 0.05 were considered significant. Statistical analysis was performed using SPSS software version 16.0 (IBM, Chicago, IL, USA).

## 5. Conclusions

In conclusion, we described the expression profile of lncRNAs and mRNAs in NPC metastasis by microarray and found several hundred dysregulated lncRNAs and mRNAs. After qRT-PCR verification, we explored the biological function of the most abnormally expressed lncRNA, ENST00000470135. The results of in vitro functional experiments suggest that the lncRNA promote migration, invasion and proliferation of NPC cells. This investigation to elucidate the roles of lncRNAs in NPC metastasis provides novel insight into the molecular mechanisms of disease progression, which may have significance for NPC therapy. In the near future, we intend to study the value of the dysregulated lncRNAs identified in this study as prognostic biomarkers using a large number of patient samples, and also explore their biological function and regulation mechanism, with the overall objective of providing novel and effective therapeutic strategies for NPC.

## Figures and Tables

**Table 1:** Twenty-six significantly differentially expressed long non-coding RNAs (lncRNAs) in nasopharyngeal carcinoma (NPC) cell lines. (Seq Name: sequence name).

Seq Name	5-8F/6-10B		S18/S26		Gene Symbol	Relationship
	Absolute Fold Change	Regulation	Absolute Fold Change	Regulation		
ENST00000448897	41.825104	up	9.1273985	up	RP11-149I23.3	intronic antisense
ENST00000510469	32.153175	up	5.211069	up	CTD-2263F21.1	intronic antisense
TCONS_00023084	23.13072	up	7.283718	up	XLOC_010978	intergenic
uc.82-	18.365393	up	73.236496	up	uc.82	intergenic
ENST00000493473	16.371115	up	21.638502	up	RP11-10O22.2	intergenic
ENST00000470135	12.599286	up	7.5503197	up	RP5-884M6.1	intergenic
ENST00000531193	12.416448	up	29.618727	up	RP11-680F20.6	natural antisense
ENST00000448579	10.321822	up	7.983826	up	LINC00114	intergenic
uc010idt.2	7.7130494	up	7.294234	up	LOC152742	intergenic
NR_048550	7.642444	up	48.898594	up	LINC00210	intergenic
NR_027301	7.1894917	up	7.240037	up	LOC148189	intergenic
NR_036430	7.1285357	up	100.50338	up	SRGN	exon sense-overlapping
NR_038311	5.942556	up	6.4440546	up	ITGB2-AS1	natural antisense
NR_037676	5.785305	up	5.5476494	up	FRMD6-AS1	intronic antisense
TCONS_00011820	5.313099	up	5.635163	up	XLOC_005303	intergenic
ENST00000560097	50.478348	down	28.228682	down	RP11-499F3.2	intergenic
ENST00000567197	30.3572	down	24.57276	down	RP11-44F21.5	intergenic
ENST00000520819	21.923645	down	5.7550936	down	RP11-11N9.4	intergenic
ENST00000560054	21.549032	down	24.84218	down	RP11-499F3.2	intergenic
uc002vwe.1	13.08117	down	14.466249	down	AK023507	exon sense-overlapping
TCONS_00016513	12.171028	down	5.3345256	down	XLOC_007896	intergenic
ENST00000537269	12.126776	down	5.0430155	down	U47924.27	intergenic
NR_027242	8.920797	down	5.522551	down	SSTR5-AS1	intronic antisense
ENST00000563602	8.73123	down	8.405632	down	RP11-44F21.5	intergenic
uc022acj.1	7.0227847	down	40.83505	down	BC041623	intron sense-overlapping
uc003zlo.1	6.7758846	down	7.096536	down	AK021570	intergenic

## Supplementary Materials

Supplementary materials can be found at www.mdpi.com/1422-0067/17/11/1956/s1.

## Abbreviations

NPC	Nasopharyngeal carcinoma
lncRNA	Long non-coding RNA
LNM	Lymph node metastasis
GO	Gene ontology
BP	Biological process
CC	Cellular component
MF	Molecular function
KEGG	Kyoto Encyclopedia of Genes and Genomes
MTT	3-(4,5)-dimethylthiahiazo(-z-y1)-3,5-di-phenytetrazoliumromide
M-MLV	Moloney Murine Leukemia Virus
Seq Name	Sequence name

## References

[1] Torre L.A., Bray F., Siegel R.L., Ferlay J., Lortet-Tieulent J., Jemal A. (2015). Global cancer statistics, 2012. CA Cancer J. Clin..

[2] Liu N., Chen N.Y., Cui R.X., Li W.F., Li Y., Wei R.R., Zhang M.Y., Sun Y., Huang B.J., Chen M. (2012). Prognostic value of a microRNA signature in nasopharyngeal carcinoma: A microRNA expression analysis. Lancet Oncol..

[3] Ng W.T., Choi C.W., Lee M.C., Chan S.H., Yau T.K., Lee A.W. (2009). Familial nasopharyngeal carcinoma in hong kong: Epidemiology and implication in screening. Fam. Cancer.

[4] Feng B.J., Huang W., Shugart Y.Y., Lee M.K., Zhang F., Xia J.C., Wang H.Y., Huang T.B., Jian S.W., Huang P. (2002). Genome-wide scan for familial nasopharyngeal carcinoma reveals evidence of linkage to chromosome 4. Nat. Genet..

[5] Sarmiento M.P., Mejia M.B. (2014). Preliminary assessment of nasopharyngeal carcinoma incidence in the philippines: A second look at published data from four centers. Chin. J. Cancer.

[6] Lai S.Z., Li W.F., Chen L., Luo W., Chen Y.Y., Liu L.Z., Sun Y., Lin A.H., Liu M.Z., Ma J. (2011). How does intensity-modulated radiotherapy versus conventional two-dimensional radiotherapy influence the treatment results in nasopharyngeal carcinoma patients?. Int. J. Radiat. Oncol. Biol. Phys..

[7] Chen Y., Sun Y., Liang S.B., Zong J.F., Li W.F., Chen M., Chen L., Mao Y.P., Tang L.L., Guo Y. (2013). Progress report of a randomized trial comparing long-term survival and late toxicity of concurrent chemoradiotherapy with adjuvant chemotherapy versus radiotherapy alone in patients with stage III to IVB nasopharyngeal carcinoma from endemic regions of China. Cancer.

[8] Mehlen P., Puisieux A. (2006). Metastasis: A question of life or death. Nat. Rev. Cancer.

[9] Gibb E.A., Brown C.J., Lam W.L. (2011). The functional role of long non-coding RNA in human carcinomas. Mol. Cancer.

[10] Hirata H., Hinoda Y., Shahryari V., Deng G., Nakajima K., Tabatabai Z.L., Ishii N., Dahiya R. (2015). Long noncoding RNA MALAT1 promotes aggressive renal cell carcinoma through Ezh2 and interacts with miR-205. Cancer Res..

[11] Yuan J.H., Yang F., Wang F., Ma J.Z., Guo Y.J., Tao Q.F., Liu F., Pan W., Wang T.T., Zhou C.C. (2014). A long noncoding RNA activated by TGF-β promotes the invasion-metastasis cascade in hepatocellular carcinoma. Cancer Cell.

[12] Xu T.P., Liu X.X., Xia R., Yin L., Kong R., Chen W.M., Huang M.D., Shu Y.Q. (2015). SP1-induced upregulation of the long noncoding RNA TINCR regulates cell proliferation and apoptosis by affecting KLF2 mRNA stability in gastric cancer. Oncogene.

[13] Geisler S., Coller J. (2013). RNA in unexpected places: Long non-coding RNA functions in diverse cellular contexts. Nat. Rev. Mol. Cell Biol..

[14] Roberts T.C., Morris K.V., Weinberg M.S. (2014). Perspectives on the mechanism of transcriptional regulation by long non-coding RNAs. Epigenetics.

[15] Wang K.C., Chang H.Y. (2011). Molecular mechanisms of long noncoding RNAs. Mol. Cell.

[16] Lu Y., Li T., Wei G., Liu L., Chen Q., Xu L., Zhang K., Zeng D., Liao R. (2016). The long non-coding RNA NEAT1 regulates epithelial to mesenchymal transition and radioresistance in through miR-204/ZEB1 axis in nasopharyngeal carcinoma. Tumour Biol..

[17] Zhuang K., Wu Q., Jin C.S., Yuan H.J., Cheng J.Z. (2016). Long non-coding RNA HNF1A-AS is upregulated and promotes cell proliferation and metastasis in nasopharyngeal carcinoma. Cancer Biomark..

[18] Jin C., Yan B., Lu Q., Lin Y., Ma L. (2016). The role of MALAT1/miR-1/slug axis on radioresistance in nasopharyngeal carcinoma. Tumour Biol..

[19] Nie Y., Liu X., Qu S., Song E., Zou H., Gong C. (2013). Long non-coding RNA hotair is an independent prognostic marker for nasopharyngeal carcinoma progression and survival. Cancer Sci..

[20] Zhang W., Huang C., Gong Z., Zhao Y., Tang K., Li X., Fan S., Shi L., Li X., Zhang P. (2013). Expression of LINC00312, a long intergenic non-coding RNA, is negatively correlated with tumor size but positively correlated with lymph node metastasis in nasopharyngeal carcinoma. J. Mol. Histol..

[21] Tu L.X., Fang W.Y., Liu Z., Li X., He Y., Xie S.M., Yao K.T. (2009). Establishment of a stable nasopharyngeal carcinoma cell line with lentivirus-mediated RNA interference for EIF4G1 gene silencing. Nan Fang Yi Ke Da Xue Xue Bao.

[22] Li X.J., Ong C.K., Cao Y., Xiang Y.Q., Shao J.Y., Ooi A., Peng L.X., Lu W.H., Zhang Z., Petillo D. (2011). Serglycin is a theranostic target in nasopharyngeal carcinoma that promotes metastasis. Cancer Res..

[23] Li G.P., Wang H., Lai Y.K., Chen S.C., Lin M.C., Lu G., Zhang J.F., He X.G., Qian C.N., Kung H.F. (2011). Proteomic profiling between CNE-2 and its strongly metastatic subclone S-18 and functional characterization of HSP27 in metastasis of nasopharyngeal carcinoma. Proteomics.

[24] Gene Ontology Consortium (2015). Gene ontology consortium: Going forward. Nucleic Acids Res..

[25] Ou S.H., Zell J.A., Ziogas A., Anton-Culver H. (2007). Epidemiology of nasopharyngeal carcinoma in the united states: Improved survival of chinese patients within the keratinizing squamous cell carcinoma histology. Ann. Oncol..

[26] Cao S.M., Simons M.J., Qian C.N. (2011). The prevalence and prevention of nasopharyngeal carcinoma in China. Chin. J. Cancer.

[27] Yang F., Zhang L., Huo X.S., Yuan J.H., Xu D., Yuan S.X., Zhu N., Zhou W.P., Yang G.S., Wang Y.Z. (2011). Long noncoding RNA high expression in hepatocellular carcinoma facilitates tumor growth through enhancer of zeste homolog 2 in humans. Hepatology.

[28] Li X., Lin Y., Yang X., Wu X., He X. (2016). Long noncoding RNA H19 regulates EZH2 expression by interacting with miR-630 and promotes cell invasion in nasopharyngeal carcinoma. Biochem. Biophys. Res. Commun..

[29] Bo H., Gong Z., Zhang W., Li X., Zeng Y., Liao Q., Chen P., Shi L., Lian Y., Jing Y. (2015). Upregulated long non-coding RNA AFAP1-AS1 expression is associated with progression and poor prognosis of nasopharyngeal carcinoma. Oncotarget.

[30] Gao W., Chan J.Y., Wong T.S. (2014). Differential expression of long noncoding RNA in primary and recurrent nasopharyngeal carcinoma. BioMed Res. Int..

[31] Yang Q.Q., Deng Y.F. (2015). Genome-wide analysis of long non-coding RNA in primary nasopharyngeal carcinoma by microarray. Histopathology.

[32] Zhang B., Wang D., Wu J., Tang J., Chen W., Chen X., Zhang D., Deng Y., Guo M., Wang Y. (2016). Expression profiling and functional prediction of long noncoding RNAs in nasopharyngeal nonkeratinizing carcinoma. Discov. Med..

[33] Liu T.H., Zheng F., Cai M.Y., Guo L., Lin H.X., Chen J.W., Liao Y.J., Kung H.F., Zeng Y.X., Xie D. (2016). The putative tumor activator ARHGEF3 promotes nasopharyngeal carcinoma cell pathogenesis by inhibiting cellular apoptosis. Oncotarget.

[34] Hui K.F., Lam B.H., Ho D.N., Tsao S.W., Chiang A.K. (2013). Bortezomib and SAHA synergistically induce ROS-driven caspase-dependent apoptosis of nasopharyngeal carcinoma and block replication of Epstein-Barr virus. Mol. Cancer Ther..

[35] Lu Z.X., Ma X.Q., Yang L.F., Wang Z.L., Zeng L., Li Z.J., Li X.N., Tang M., Yi W., Gong J.P. (2008). Dnazymes targeted to EBV-encoded latent membrane protein-1 induce apoptosis and enhance radiosensitivity in nasopharyngeal carcinoma. Cancer Lett..

[36] Yang C.F., Peng L.X., Huang T.J., Yang G.D., Chu Q.Q., Liang Y.Y., Cao X., Xie P., Zheng L.S., Huang H.B. (2014). Cancer stem-like cell characteristics induced by EB virus-encoded LMP1 contribute to radioresistance in nasopharyngeal carcinoma by suppressing the p53-mediated apoptosis pathway. Cancer Lett..

[37] Crook T., Nicholls J.M., Brooks L., O‘Nions J., Allday M.J. (2000). High level expression of ΔN-p63: A mechanism for the inactivation of p53 in undifferentiated nasopharyngeal carcinoma (NPC)?. Oncogene.

[38] Chen C.C., Liu H.P., Chao M., Liang Y., Tsang N.M., Huang H.Y., Wu C.C., Chang Y.S. (2014). NF-κB-mediated transcriptional upregulation of TNFAIP2 by the Epstein-Barr virus oncoprotein, LMP1, promotes cell motility in nasopharyngeal carcinoma. Oncogene.

[39] Niemhom S., Kitazawa S., Murao S., Kunachak S., Maeda S. (2000). Co-expression of p53 and BCL-2 may correlate to the presence of Epstein-Barr virus genome and the expression of proliferating cell nuclear antigen in nasopharyngeal carcinoma. Cancer Lett..

[40] Yates A., Akanni W., Amode M.R., Barrell D., Billis K., Carvalho-Silva D., Cummins C., Clapham P., Fitzgerald S., Gil L. (2016). Ensembl 2016. Nucleic Acids Res..

[41] Qi P., Zhou X.Y., Du X. (2016). Circulating long non-coding RNAs in cancer: Current status and future perspectives. Mol. Cancer.

[42] Dong L., Qi P., Xu M.D., Ni S.J., Huang D., Xu Q.H., Weng W.W., Tan C., Sheng W.Q., Zhou X.Y. (2015). Circulating CUDR, LSINCT-5 and PTENP1 long noncoding RNAs in sera distinguish patients with gastric cancer from healthy controls. Int. J. Cancer.

[43] Su Y.J., Yu J., Huang Y.Q., Yang J. (2015). Circulating long noncoding RNA as a potential target for prostate cancer. Int. J. Mol. Sci..

[44] Fayda M., Isin M., Tambas M., Guveli M., Meral R., Altun M., Sahin D., Ozkan G., Sanli Y., Isin H. (2016). Do circulating long non-coding RNAs (lncRNAs) (LincRNA-p21, GAS 5, HOTAIR) predict the treatment response in patients with head and neck cancer treated with chemoradiotherapy?. Tumour Biol..

[45] Livak K.J., Schmittgen T.D. (2001). Analysis of relative gene expression data using real-time quantitative PCR and the 2^−ΔΔ*C*t^ method. Methods.

